# Endomyocardial biopsy via the femoral access - still safe and valuable diagnostic tool

**DOI:** 10.1186/s12872-016-0406-0

**Published:** 2016-11-15

**Authors:** Sylwia Sławek, Aleksander Araszkiewicz, Agnieszka Gaczkowska, Justyna Koszarska, Damian Celiński, Marek Grygier, Maciej Lesiak, Stefan Grajek

**Affiliations:** Department of Cardiology, Poznan University of Medical Sciences, Poznan, Poland

**Keywords:** Endomyocardial biopsy, Complications, Heart transplantation, Myocarditis, Pericardial tamponade

## Abstract

**Background:**

The endomyocardial biopsy has proven to be an integral diagnostic tool for surveillance of cardiac allograft rejection and identification of myocardial diseases. Nevertheless, this invasive procedure is not risk-free. This study focuses on the risk of complications and diagnostic performance of right ventricular endomyocardial biopsy (EMB).

**Methods:**

In this single-center retrospective study, we analyzed 315 EMB procedures performed between July 2008 and May 2015 in 73 patients. All EMBs were made via the right femoral vein approach under fluoroscopic control to evaluate suspected myocarditis, unclear heart failure, unexplained cardiomyopathy, assumed infiltrative and storage disease or as a part of routine allograft rejection monitoring and clinically suspected rejection diagnosis after heart transplantation (HTx). Obtained specimens were diagnosed histopathologically by one experienced pathologist. All patients underwent a 12-lead electrocardiogram (ECG), ECG monitoring, transthoracic echocardiography before and after EMB to obtain a detailed assessment of the incidence of heart rhythm disorders, pericardial effusions or worsening valve insufficiency. Complications resulting from the procedure were classified as major or minor according to the risk of death.

**Results:**

Among all the 315 biopsies, 86.67% were performed in 32 patients after HTx, 3.81% in patients with myocarditis, 2.54% in patients with dilated cardiomyopathy and 1.9% in patients with amyloidosis. The overall complications rate was 1.9% (6 of 315 procedures). Major complications included perforation with pericardial tamponade requiring surgical intervention (0.64%, 2 of 315 procedures). Minor complications included: pericardial effusion (0.32%, 1 of 315 procedures), local hematoma (0.64%, 2 of 315 procedures) and right coronary artery-right ventricle fistula in HTx recipient (0.32%, 1 of 315 procedures).

**Conclusions:**

EMB is a safe procedure with low risk of serious complications and high effectiveness for the evaluation of unexplained left ventricle dysfunction and monitoring allograft rejection after HTx.

## Background

The endomyocardial biopsy (EMB) constitutes an established tool for surveillance of cardiac allograft rejection and identification of myocardial diseases [1]. EMB plays a pivotal role in diagnosis of myocarditis, infiltrative or storage myocardial disorders and monitoring of heart transplant rejection. EMB is also indicated in detecting of cardiac tumors, cardiac toxicity as well as ventricular arrhythmias [2]. In recent years, the utility and accuracy of EMB is increasingly growing due to application of new research tools including immunohistochemistry and molecular biology techniques in identification of cardiac diseases [3, 4]. EMB is most commonly performed through the femoral or jugular vein access under the guidance of fluoroscopy. The appropriate number samples (usually 5–10) are generally obtained from interventricular septum (IVS) [5]. However, samples may also be collected from the free wall of the right or left ventricle (LV) [6]. Although available data indicate that complication rate is not high, this procedure is associated with potentially serious complications. It stands to reason that lowering the incidence of critical complications is a major concern in performing EMB.

The aim of the present study was to evaluate clinical and histopathological findings of EMBs. We also assessed safety of EMB through the description and quantification of the procedure related complications.

## Methods

All consecutive patients who underwent EMB between July 2008 and May 2015 at the Department of Cardiology, Poznan, Poland were included in this retrospective analysis. Bioethics Committee at the Poznan University of Medical Sciences approved the study. Authors obtained written consent from all patients participating in the study.

Biopsies were performed according to current guidelines as a tool for the diagnostic evaluation of suspected myocarditis, unclear heart failure, unexplained cardiomyopathy, assumed infiltrative and storage disease or as a part of routine allograft rejection monitoring and clinically suspected rejection diagnosis in heart transplant recipients [2].

In patients who underwent heart transplantation (HTx), EMB procedures were made in accordance with a current protocol: a week after HTx, every 2 weeks for the next 8 weeks, once for the next 4 weeks, once for the next 6 weeks, then every 3 months for the next two years, and afterwards every 12 months for the next years [7]. The two nondiagnostic myocardial specimens were excluded from the study.

### Endomyocardial biopsy procedure

**Fig. 1:**
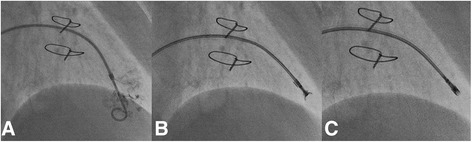
Endomyocardial biopsy (EMB) of right ventricle (RV) “step-by-step”: **a** Confirmation of the position of 7F curved sheath inside RV with the use of pig-tail catheter and small amount contrast injection. **b** Biopsy forceps opened and **c** closed. 5–10 samples of myocardium are usually taken during the procedure

All EMB procedures were performed via the right femoral vein access under fluoroscopic guidance. In some cases additionally 2D-echocardiography support was used. A 7F long, curved sheath (96 cm, Cordis) was placed in the right ventricle (RV) and the bioptome was used to collect specimens. 6 ± 2 myocardial tissue samples, 1–2 mm in the diameter were harvested from the apical segment of the right side of IVS (Fig. 1). All EMB procedures were limited to the two experienced operators. Harvested myocardial tissue specimens were fixed in 10% buffered formalin and then sent for further histopathological evaluation. Additional samples were collected on 0.9% saline or immediately frozen in optimum cutting temperature compound with watersoluble glycols cooled in liquid nitrogen and stored at −80 °C. For transmission electron microscopy, EMB samples were fixed in 2% glutaraldehyde in 0.1 mol/L phosphate buffer (pH 7.3).

In addition to continuous electrocardiographic monitoring, right atrial pressure and right ventricular (RV) pressures were recorded before as well as promptly after sampling to detect potentially impending pericardial tamponade. Moreover, a 12-lead electrocardiogram (ECG), ECG monitoring, transthoracic echocardiography before and immediately after as well as 12 h after EMB procedures were assessed to obtain a detailed evaluation of the incidence of conduction abnormalities, arrhythmias, pericardial effusions and worsening valve insufficiency.

### Histopathological assessment

Histological evaluation was performed by one experienced pathologist. The diagnosis of allograft rejection was performed according to the revised International Society for Heart and Lung Transplantation (ISHLT) classification [8]. In suspicion of myocarditis, the diagnosis was confirmed based on the histopathological Dallas criteria, immunochemistry and viral genome analysis results [9]. The diagnosis of specific type of cardiomyopathy was made in accordance with the recommendations of American Heart Association/European Society of Cardiology [10, 11]. Furthermore, the presence of well-formed granulomas with negative stains for microorganisms were the criteria used for the confirmation of sarcoidosis. The identification of infiltrative amyloidosis disease was reached by use Congo red stains and electron microscopy. Cardiac toxicity was diagnosed in the presence of loss of myofibrils, increased fibrosis and vacuolization of the cytoplasm.

### Complications classification

Complications resulting from the procedure were classified as major or minor according to the risk of death. Definition and assessment of major and minor EMB complications were in accordance with previous studies [12, 13]. Major complications were defined as: death, pericardial tamponade, hemo- and pneumopericardium, permanent atrioventricular block requiring pacemaker implantation, myocardial infarction, transient cerebral ischemic attack and stroke, severe tricuspid valve damage, while minor complications included transient chest pain, access site hematomas, transient arrhythmias, transient hypotension, and small pericardial effusions.

### Statistical Analysis

Continuous variables were expressed as mean ± standard deviation (SD). Categorical variables were presented as absolute values and percentages. Statistical analyses were performed using Microsoft Excel Office 2011 software (Microsoft Corporation, Redmond, Wash).

## Results

A total of 315 EMB procedures were performed among 73 patients. The majority of recorded patients were men (57 of 73, 78.1%). The mean age of patients was 47.6 ± 12.1 years (range: 18–68 years). The detailed baseline patient characteristics are present in Table 1.

**Table 1:** Baseline patients characteristics

Baseline characteristics	All patients (*n* = 73)	HTx recipients (*n* = 32)	Other patients (*n* = 41)
Age (years)	47.6 ± 12.1	48.91 ± 11.24	40.02 ± 14.53
Sex			
Females	16	9	7
Males	57	23	32
BMI (kg/m^2)^	24.37 ± 4.11	24.31 ± 3.99	24.05 ± 5.43
NYHA Class			
I, II,	35	31	4
III, IV	39	1	38
Coronary artery disease	3	3	0
Myocardial infarction	0	0	0
Hypertension	11	11	0
Diabetes mellitus	9	9	0
Prior pacemaker implantation	2	2	0
Prior cardioverter-defibrilator implantation	0	0	0
Prior cardiac resynchronisation therapy device implantation	0	0	0
Prior rheumatic disease	1	1	0
Renal failure	14	9	5
GFR (ml/min/m^2^)	71.66 ± 23.48	53.2 ± 23.16	72.83 ± 12.17
Blood pressure (mmHg)			
Systolic	127.58 ± 15.29	129.89 ± 33.3	99.00 ± 12.04
Diastolic	81.38 ± 11.23	82.95 ± 9.54	81.38 ± 11.23
Electrocardiogram			
Sinus rhytm	70	30	42
Atrioventricular block	0	0	0
Left Bundle Branch Block	4	2	2
Right Bundle Branch Block	6	6	0
Heart rate on admission (min^−1)^	95.26 ± 13.48	95.98 ± 13.37	84.60 ± 12.18
PQ-interval (s)	0.147 ± 0.043	0.160 ± 0.06	0.14 ± 0.06
QRS-width (s)	0.119 ± 0.022	0.120 ± 0.06	0.18 ± 0.018
QT-interval (s)	0.36 ± 0.32	0.38 ± 0.14	0.36 ± 0.50
ST-segment alterations	5	2	3
Negative T-wave	5	2	3
Cardiac Biomarkers			
Creatine kinase (U/L)	85.78 ± 82.17	38.26 ± 75.68	187.00 ± 192.85
Creatine kinase- MB (U/L)	21.44 ± 8.64	21.27 ± 8.86	16.2 ± 15.81
Troponin I (ng/mL)	0.37 ± 1.67	0.23 ± 0.97	4.51 ± 2.75
Brain natriuretic peptide (pg/mL)	312.08 ± 365.13	261.13 ± 284.35	948.02 ± 641.85
Laboratory results on admission			
C-reactive protein (mg/L)	32.29 ± 8.72	12.21 ± 34.35	47.18 ± 62.20
Hemoglobin concentatration (mmol/L)	7.8 ± 1.2	7.61 ± 1.04	10.35 ± 0.67
White blood cell count (1 × 10^9^/L)	6.47 ± 3.44	6.23 ± 3.19	10.23 ± 5.41
Platelets count (1 × 10^9^/L)	107.78 ± 57.27	205.13 ± 56.97	215.83 ± 55.42
Echocardiography			
Ejection fraction (%)	61.24 ± 14.65	64.32 ± 8.92	17.67 ± 5.16
Left ventricle end diastolic diameter (mm)	45.92 ± 14.65	44.41 ± 4.58	68.33 ± 10.04
Interventricular septum (mm)	11.9 ± 2.14	12.00 ± 2.07	9.91 ± 2.20
Posterior wall (mm)	11.38 ± 2.73	11.42 ± 2.73	10.75 ± 2.98
Right ventricle end diastolic diameter (mm)	28.19 ± 4.23	27.98 ± 4.13	32.00 ± 4.20
Right ventricular systolic pressure (mmHg)	32.39 ± 8.72	32.27 ± 9.00	33.25 ± 4.71
Pericardial effusion			
< 4 mm	6	4	2
> 4 mm	0	0	0
Tricuspid valve dysfunction			
Mild	5	5	2
Moderate	3	1	6
Severe	0	0	2
Cardiac catheterization			
Right atrial pressure (mmHg)			
Systolic	14.00 ± 7.18	12.00 ± 6.00	16.00 ± 9.00
Diastolic	8.00 ± 5.30	6.00 ± 3.50	10.00 ± 7.00
Mean	11 ± 6.14	8.00 ± 2.70	14.00 ± 8.00
Right ventricular pressure (mmHg)			
Systolic	39.2 ± 17.73	27.00 ± 6.00	51.00 ± 18.4
Diastolic	7.33 ± 8.55	6.00 ± 4.00	8.00 ± 9.00
End diastolic	10 ± 6.83	4.00 ± 5.50	15.00 ± 5.00
Pulmonary Artery Pressure (mmHg)			
Systolic	31.00 ± 11.64	24.00 ± 7.00	39.00 ± 11.00
Diastolic	16.33 ± 9.91	12.00 ± 5.00	20.00 ± 13.00
Mean	19.30 ± 13.70	15 ± 7.00	30.00 ± 16.50

Continuous variables are presented as mean ± SD. Categorical variables are presented as absolute values (n=). *NYHA* New York Heart Association

Among all the 315 biopsies, 86.67% were EMB procedures performed in patients after HTx. Myocarditis was the second most frequently diagnosed condition with the incidence of 3.81%. It was followed by dilated cardiomyopathy that reached 2.54%. Amyloidosis was diagnosed with a relatively high incidence in 1.9% of EMB procedures. Drug-induced cardiomyopathy, cardiac tumor and peripartum cardiomyopathy were the rarest identified entities, with the incidence of 0.32%. Table 2 lists the detailed frequency of each diagnosed cardiac disorder.

**Table 2:** The frequency of cardiac disorders diagnosed using endomyocardial biopsy (EMB)

Diagnosis	Number of EMB procedures	% of EMB procedures
Myocarditis	12	3.81
Inflammatory cardiomyopathy	5	1.59
Dilated cardiomyopathy	8	2.54
Hypertrophic cardiomyopathy	4	1.27
Peripartum cardiomyopathy	1	0.32
Drug-induced cardiomyopathy	1	0.32
Cardiac toxicity	2	0.64
Cardiac tumor	1	0.32
Sarcoidosis	2	0.64
Amyloidosis	6	1.9
Heart transplant state	273	86.67

Among the 273 EMB procedures performed in 32 HTx recipients, moderate rejection (grade 2a) was detected in 7 biopsy samples (2.56%), while mild (grade 1a) was observed in 56 samples (20.5%). 210 samples (76.9%) did no show evidence of cellular allograft rejection. Moreover, all moderate rejections were detected during first 24 months after HTx, and 3 of 4 moderate rejections occurred in the first 6 months after HTx.

**Fig. 2:**
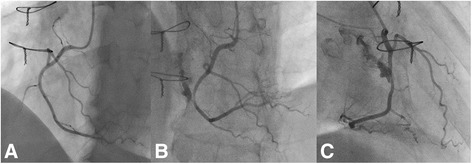
Right coronary artery to right ventricle fistula after endomyocardial biopsy in a patient after heart transplantation. **a** Angiography of right coronary artery in a patient 3 months after HTX – no signs of fistula. **b** Coronary angiography directly following EMB 12 months after HTX. Well visible fistula to right ventricle. *Left* anterior oblique projection. **c** *Right* anterior oblique (RAO) projection

The overall complications rate was 1.9% (6 of 315 procedures). Major complications included 2 cases (0.64%) of perforation requiring surgical intervention. There was no death associated with the EMB procedure. Minor complications included one case of pericardial effusion, 2 cases of local hematoma and one case of right coronary artery-right ventricular fistula in HTx recipient (Fig. 2) (See Table 3). Table 4 presents the detailed characteristics of patients with EMB complications. The coronary artery fistula was asymptomatic and small in size. It was diagnosed in the TTE and annual coronary arteriography. There was no difference in the complication rate between the two operators, each of them performed one EMB procedure complicated by tamponade.

**Table 3:** The frequency of endomyocardial biopsy (EMB) complications

Complications	Number of events	% of events
All complications	6	1.9
Major complications	2	0.64
Perforation with pericardial tamponade	2	0.64
Minor complications	4	1.28
Pericardial effusion	1	0.32
Access site hematoma	2	0.64
Right coronary artery-right ventricular fistula	1	0.32
Without complications	309	98.1

## Discussion

Since the introduction of catheter-based myocardial biopsy by Sakakibara and Konno in 1962, EMB has been considered a valuable tool for evaluation of cardiac tissue for cardiomyopathy, myocarditis, unexplained arrhythmia, cardiac tumor, cardiac involvement of systemic disease and cardiac allograft rejection. Available data indicate that EMB is generally safe procedure, with relatively low complications rate. Possible major complications associated with EMB procedures include: pericardial effusion, pericardial tamponade requiring pericardiocentesis, hemo- and pneumothorax, myocardial infarction, transient ischemic attack, stroke, permament complete atrioventricular block, severe tricuspid regurgitation or death. As minor complications are reported: small pericardial effusions, transient chest pain, transient arrhythmias, hypotension, local nerve paresis, local hematoma, and femoral arterial-venous fistula [6].

**Table 4:** The detailed characteristics and clinical course of patients with endomyocardial biopsy (EMB) complications

Patient	Sex	Age (years)	NYHA Class on admission	Diagnosis	Type of complication	TTE after EMB	Symptoms of developing complication	Method of complication treatment	Recovery
1	Male	28	IV	Myocarditis	Tamponade	15 mm of fluid in pericardium	Chest pain, sudden drop in blood pressure, tachycardia	Immediate partial sternotomy, hematoma decompression and suturing of damaged right venticle	II NYHA class at discharge
2	Female	49	IV	Myxoma in the right ventricular outflow tract	Tamponade	25 mm of fluid in pericardium	Chest pain, sudden drop in blood pressure, tachycardia	Immediate sternotomy, hematoma decompression, tumor removal	Full
3	Male	32	II	Pericardial effusion	Pericardial effusion	Up to 6 mm of fluid in pericardium	Without symptoms	Diuretics	Full
4	Male	57	II	EMB - a year after HTx	Access site hematoma	Without fluid/tricuspid insufficiency	Puncture site pain	Conservative	Full
5	Female	49	II	EMB −2 months after HTx	Access site hematoma	Without fluid/tricuspid insufficiency	Puncture site pain	Conservative	Full
6	Male	60	I	EMB – 3 years after HTx	RCA-RV fistula	Diastolic jet to the lumen of RV (the diagnosis was made on the basis of coronarography	Asymptomatic	Conservative -“watchful waiting”	Full

*NYHA* New York Heart Association, *RCA* right coronary artery, *RV* right ventricle

The present study confirmed the safety of EMB. The cumulative complication rate was 1.9%. In the literature EMB- related complications rate varies from 0.71% to 9.2% [14, 15]. In previous studies major complications was reported in less than 1.5% of performed procedures, while minor complications ranged between 1.0% and 7.9% [15, 16]. See Table 5.

**Table 5:** Risk of endomyocardial biopsy (EMB) complications reported in previous studies

Author (year)	Number of biopsies	Overall complications rate (%)	Major complications rate	Minor complications rate	Reference
Deckers (1992)	546	6	1.2^a^	4.8^a^	[24]
Hiramitasu (1998)	19 964		0.7		[25]
Felker (1999)	323		0.32		[26]
Holzmann (2008)	3048	1.25^a^	0.1^a^	1.15^a^	[12]
Yilmaz (2010)	622 (LVEMB)	3.5 (LVEMB)	0.6 (LVEMB)	2.9 (LVEMB)	[13]
	490 (RVEMB)	5.9 (RV EMB)	0.8 (RVEMB)	5.1 (RVEMB)	
Huang (2010)	439	1.61	0.0	1.61	[27]
Saraiva (2011)	2217	0.71	-	-	[14]
Fiorelli (2012)	5347	6.2	0.3	5.9	[28]
Bennet (2013)	851	1.9	0.9	1.0	[16]
Jang (2013)	228	9.2	1.3	7.9	[15]
Chimenti (2013)	3549 (LVEMB)	2.33 (LVEMB)	0.33 (LVEMB)	2.0 (LVEMB)	[22]
	3068 (RVEMB)	1.8 (RVEMB)	0.45 (RVEMB)	1.35 (LVEMB)	
Strecker (2013)	1896		1.0		[21]
Isogai (2015)	9508		0.98		[19]
Schulz (2015))~	37	2.7	0	2.7	[1]
Schäufele (2015)~	41	0	0	0	[23]

^a^values calculated on the basis of the manuscript data; LVEMB- left ventricle endomyocardial biopsy; RVEMB-right ventricle endomyocardial biopsy; ~ - transradial approach

In our study the risk of major complications was low - 0.64%. The only serious complication among our patients was iatrogenic cardiac perforation with subsequent tamponade requiring surgical intervention. Pericardial tamponade occurred in 2 patients (0.64% of all EMB procedures), in one patient with myocarditis and in another one with tumor in the right ventricular outflow tract. One patient developed asymptomatic pericardial effusion not progressed to cardiac tamponade.

One of patient after HTx developed right coronary artery to right ventricle fistula. It was reported that repeated EMBs in heart transplant recipients are directly related to the incidence coronary arteries fistulas [17]. Nevertheless, there was no association between the number of performed EMBs in HTX recipients with and without coronary artery fistula [18]. The great majority of coronary artery fistulas are benign condition. In the literature, there is described the relatively high rate of spontaneous closure of fistula, what favors a conservative “watchful waiting “approach [14–18]. Due to a small size and asymptomatic course of the right coronary artery to right ventricle fistula in our patient conservative approach was adopted.

All of the EMB procedures were performed by two highly experienced interventional cardiologists. This findings support results of recent studies that the incidence of major complication was lower when EMB was performed by experienced operators [12]. Isogai *et al*. found that higher hospital volume was associated with lower rates of major complications after EMB. Pericardiocentesis was needed in 0.4% in the low-volume hospitals, in 0.2% in medium-volume hospitals and in 0.1% in high-volume hospitals, respectively (p = 0.019). The rates of temporary pacing also were lower in the high-volume hospitals (0.2%) in comparison to the low-volume (1.0%) and medium-volume hospitals (0.7%) (*p* < 0.001) [19].

It is also known that the frequency of complications, especially minor is associated with operative technique, heart disease and quality of patients monitoring. In this work all biopsies were performed via femoral vein access that allows avoiding the risk of pneumothorax or hemothorax related to internal jugular venous access. However, femoral venous access is associated with an increased risk of a deep vein thrombosis and pulmonary embolism because of the obligate immobilization and application of longer sheaths. Recently, Imanura *et al*. described that internal jugular access was associated with lower operation and radiation exposure times, and lower radiation exposure dose and contrast usage compared with the femoral approach [20]. Nevertheless, the full interpretation of these results is limited by the retrospective nature of the study, lack of the randomization, biased patients allotments to applied technique.

What is more, Imanura *et al*. reported that internal jugular vein approach had less overall complications rate than the femoral vein approach 2.7% versus 10.0% (*p* = 0.011). All complications were transient, but only internal jugular vein approach was complicated by transient neurologic events in 0.9% (3 of 329 EMBs). Moreover, only femoral vein access was complicated by nonsustained ventricular tachycardia 4% (2 of 50 EMBs) and transient bundle branch block 4% (2 of 50 EMBs), whereas internal jugular vein access was complicated by atrial tachyarrhythmia 0.9% (3 of 329 EMBs) [20]. In the study of Strecker *et al*. major complications after EMB performed through the right internal jugular vein occured in 1.0% of EMBs (19 of 1896 EMBs). Twelve patients developed moderate or severe tricuspid regurgitation with increased pulmonary artery systolic pressure and six of these patients received mechanical or biological tricuspid valve prosthesis. In six other patients echocardiography showed pericardial effusions around the right and left ventricle with signs of tamponade, these patients underwent pericardiocentesis on the same day. The another one patient developed supraventricular tachycardia during the biopsy, but remained asymptomatic [21]. Although small discrepancies, the results these studies indicate that the incidence of major complication was similar when using the femoral vein or jugular vein access.

Interestingly, last findings revealed that left ventricular endomyocardial biopsy (LVEMB) is as safe as right ventricular EMB. Chimenti *et al*. reported low incidence of major complication after LVEMB and comparable to after RVEMB, 0.33% and 0.45%, respectively. Moreover, the risk of perforation was lower in LVEMB in comparison to RVEMB, likely due to the thinner walls of the RV, which are easier to perforate by the bioptome. In all cases of LV perforation, authors found significant dilatation [13]. Among 755 patients with myocarditis or dilated cardiomyopathy the major complications after LVEMB occurred 0.64%, while after RVEMB in 0.82% [22]. Recently, Schulz *et al*. demonstrated the feasibility and safety of the transradial approach for LVEMB. EMB procedures were conducted in 37 patients. The overall complication rate was 2.7%, only one patient developed ventricular fibrillation which was terminated by external defibrillation [1]. Schäufele *et al*. included 42 patients and performed 41 transradial biopsy procedures. In one case they crossed over to femoral approach because of irreversible spasm of the right radial artery after administration of local anesthesia. They did not describe any complications after EMB, and the quality of obtained samples was good [23]. The application of transradial access may be reasonable in situations when simultaneous coronary angioplasty is desirable.

### Study limitations

The present study has several limitations. First, the present study was performed at a single center in a retrospective manner. Second, the approach point was not randomized, and selection bias existed in the present study. Third, we evaluated only the safety of EMB performed via the femoral vein access and we were not able to assess the complication rate associated with different approaches. Fourth, we acknowledge this study was limited by small numbers of patients, which make the results difficult to extrapolate to a larger population. The number of heart transplant patients was very small in this study, mainly due to severe shortage of donors in Poland and rarely performed heart transplants. Although the major complication rate in our study was similar to previous larger studies, it is of limited accuracy due to insufficient number of EMB procedures. Finally, another issue relates that we included all patients who underwent EMBs procedure, not only these with stable hemodynamics. Therefore, the results obtained in these patients may not be simply compared with other studies assesing only hemodynamically stable patients, in which EMB may have a lower complication rate.

## Conclusions

In conclusion, EMB is a safe procedure with low risk of serious complications and high effectiveness for the evaluation of unexplained left ventricle dysfunction and monitoring allograft rejection after HTx.

## Abbreviations

ECG: Electrocardiogram
EMB: Endomyocardial biopsy
HTx: Heart transplantation
IVS: Interventricular septum
LV: Left ventricle
LVEMB: Left ventricle endomyocardial biopsy
NYHA: New York Heart Association
RCA: Right coronary artery
RV: Right ventricle
RVEMB: Right ventricle endomyocardial biopsy
SD: Standard deviation
